# Recent five-year progress in the impact of gut microbiota on vaccination and possible mechanisms

**DOI:** 10.1186/s13099-023-00547-y

**Published:** 2023-06-12

**Authors:** Biqing Huang, Jianwei Wang, Lanjuan Li

**Affiliations:** 1 State Key Laboratory for Diagnosis and Treatment of Infectious Diseases, National Clinical Research Center for Infectious Diseases, Collaborative Innovation Center for Diagnosis and Treatment of Infectious Diseases, The First Affiliated Hospital, Zhejiang University school of medicine, Hangzhou, China; 2 Research Units of Infectious disease and Microecology, Chinese Academy of Medical Sciences & Peking Union Medical College, Hangzhou, China; 3 NHC Key Laboratory of Systems Biology of Pathogens and Christophe Mérieux Laboratory, Institute of Pathogen Biology, Key Laboratory of Respiratory Disease Pathogenomics, Chinese Academy of Medical Sciences & Peking Union Medical College, Beijing, China

**Keywords:** Gut microbiota, Vaccine, Immune efficacy

## Abstract

Vaccine is the most effective way to prevent the spread of communicable diseases, but the immune response induced by it varies greatly between individuals and populations in different regions of the world. Current studies have identified the composition and function of the gut microbiota as key factors in modulating the immune response to vaccination. This article mainly reviews the differences in gut microbiota among different groups of vaccinated people and animals, explores the possible mechanism of vaccine immunity affected by gut microbiota, and reviews the strategies for targeting gut microbiota to improve vaccine efficacy.

## Introduction

Vaccine is the most effective measure to prevent communicable diseases, which could significantly reduce the morbidity, severity, and mortality of diseases, as well as the use of antibiotics and the emergence of antibiotic resistance [1]. However, vaccine-induced immune responses vary widely between individuals and populations in different regions of the world [1]. Therefore, understanding the mechanism of this variation is critical to human health. Although many factors could affect the immunogenicity of a vaccine and thus its effectiveness, there is increasing evidence from clinical studies and animal models that the composition and function of the gut microbiota are key factors that regulate the immune response to vaccination [2].

Human gastrointestinal microbiota is composed of complex communities of bacteria, viruses, archaea, and fungi, which affect human health throughout the whole life by maintaining gastrointestinal homeostasis, regulating immune system development, metabolizing nutrients, and preventing pathogen colonization [3]. Gut microbiota could also act as a natural adjuvant, regulate host immune responses, and carry epitopes that are similar to vaccine antigens to induce cross-reaction and other ways to affect vaccine efficacy [1].

In this review, we summarized the evidence that the gut microbiota affected vaccine response and discussed the possible mechanisms of how the gut microbiota affected vaccine immunogenicity and provided new strategies for targeting the gut microbiota to optimize vaccine efficacy.

## Influence of gut microbiota on vaccine efficacy

The composition of the gut microbiota varies widely between individuals, which correlates with differences in vaccine immunogenicity [1]. we assessed evidence from animal models and clinical studies to show that the composition and function of the gut microbiota were factors associated with variation in vaccine response, as detailed in Table 1.

### Related studies from animals

Data from animal models suggest that gut microbiota plays an important role in modulating vaccine efficacy. For example, the gastrointestinal homeostasis of vancomycin-treated mice and rhesus monkeys was disrupted, which was associated with reduced serum levels of antigen-specific immunoglobulin (Ig) G following subsequent parenteral vaccination. Restoration of microbial diversity before vaccination could prevent vancomycin-induced hyporesponsiveness to vaccines. RNA-sequencing analysis of the small intestine, spleen, whole blood, and secondary lymphoid organs of vancomycin-treated mice revealed that the loss of Lactobacillus, Ruminococcus, and Clostridiaceae had significant effects on the immune system and correlated with mild inflammatory features [4]. Another study found that mice exposed to ampicillin and neomycin during infancy had significantly impaired antibody responses to five different live or adjuvanted vaccines used by infants around the world, and the impaired antibody responses could be rescued by fecal microbiota transplantation from age-matched ampicillin- and neomycin-non-treated mice [5]. Yitbarek A et al. found that chickens treated with an antibiotic cocktail (vancomycin and neomycin and metronidazole and amphotericin) had a similar phenomenon after being inoculated with a fully inactivated H9N2 subtype avian influenza virus vaccine: antibiotic-treatment chickens had reduced serum titers of H9N2-specific IgM and IgG, and normal antibody levels were restored after fecal microbiota transplantation from healthy chickens [6]. Nadeem, S. et al. induced intestinal dysbiosis in mice by administering a broad-spectrum antibiotic cocktail (amphotericin B and trimethoprim and polymyxin B and vancomycin and carbenicillin), and monitored the generated long-lasting memory T cells amount after vaccinating Bacillus Calmette-Guerin Vaccine (BCG), which could reflect the effect of BCG efficacy. It was found that gut dysbiosis significantly reduced the activation of CD4 + and CD8 + T cells in the lungs of mice, as well as the ratio of memory CD4 + and CD8 + T cells in the lungs and secondary lymphoid organs, and suppressed the proliferation and secretion of interferon-γ and tumor necrosis factor (TNF)-α by mycobacterium (M.) tuberculosis-specific T cells, hindered the clearance of M. tuberculosis in vaccinated mice, increasing M. tuberculosis colony-forming units in the lungs and spleen [7]. Twelve strains of lactic acid bacteria isolated from badger feces reduced the immune efficacy of BCG by inhibiting BCG-induced activation of the pro-inflammatory transcription factor NF-κB in macrophages [8]. Zhang, Y. et al. found that mice treated with a broad-spectrum antibiotic cocktail (ampicillin and metronidazole and neomycin and vancomycin) before vaccinating rabies vaccine, would decrease the serum titers of rabies virus-specific IgM and IgG, and virus-neutralizing antibody, and the amount of T follicular helper cells, germinal center B cells, and plasma cells in lymph nodes. Treatment with vancomycin alone had similar impairing effects on the humoral immune response compared with treatment with a broad-spectrum antibiotic cocktail. These studies suggest that antibiotic-driven dysbiosis of the gut microbiota suppresses the immune response to vaccination.

### Related research from humans

To explore the impact of gut microbiota on vaccine response in humans, Ghana, Pakistan, the Netherlands, India, Malawi, the United Kingdom(UK), Nicaragua, Zimbabwe, Bangladesh, and other countries studied the correlation between the abundance of certain bacterial families, genera, and species in the gut and the human immune response to vaccines. In Ghanaian and Pakistani infants following oral rotavirus vaccine (ORV), the gut microbiota of Ghanaian and Pakistani with well ORV vaccination responses was more similar to that of Dutch infants (increased abundance of Streptococcus Bovis and Proteus Bacteroidetes, and decreased abundance of Bacteroidetes), while Ghanaian infants with poor ORV vaccination responses had increased enteric Streptococcus and decreased Bacteroides. And the phage diversity and the presence of enterovirus B and multiple novel co-viruses were also inversely correlated with Ghanaian infants’ ORV seroconversion ratio [10–12]. Increased gut microbiota diversity was inversely associated with ORV immunogenicity in infants from India and Malawi, who had significantly lower rates of rotavirus shedding and seroconversion than those in the UK [13]. In Nicaragua, infants who responded to ORV had a higher abundance of Proteus and E. fergusonii, whereas non-responders had a higher abundance of Fusobacteria and Enterobacteriaceae [14]. Polymorpha was the only species associated with serum anti-rotavirus IgA titers in rural Zimbabwe [15]. Similar findings have been made in other vaccine studies that the gut microbiota influences vaccine efficacy. A study in Bangladeshi infants showed that the abundance of gut actinomycetes, especially Bifidobacterium longum subspecies, was associated with the high immune responses of oral polio vaccine as well as parenteral vaccines such as BCG, tetanus toxoid vaccine, and the hepatitis B virus vaccine, which manifested as positively correlated with the T cell response, and the number of CD4 + T cells, and the serum titers of vaccine-specific IgG and IgA after vaccinating 2 years, while the low vaccine responses were associated with the high abundance of Clostridium, Enterobacter and Pseudomonas spp [16]. This indicates that the gut microbiota is not only related to the immune response to the vaccine but also associated with the persistent immune response induced by the vaccine. A clinical cohort study from New Hampshire about the tetanus toxoid vaccine found that the relative abundance of Bifidobacteriaceae was inversely correlated with the specific antibody response induced by the tetanus toxoid vaccine, whereas the CDP-diacylglycerol biosynthetic pathway-related species abundance was positively correlated with specific antibody responses induced by tetanus toxoid vaccine [17]. Recently, a study about the SARS-COV-2 vaccine found that the immune response of CoronaVac recipients was significantly lower than that of the BNT162b2. Recipients with high serum titers of neutralizing antibodies to the CoronaVac had abundant Bifidobacterium, while recipients with high serum titers of neutralizing antibodies to the BNT162b2 were rich in bacteria with flagella and fimbriae in the gut. Recipients with fewer adverse events after vaccinating any of the two vaccines were enriched with large amounts of Copriprevoria and two Megamonas [18]. In addition, Tang, B. et al. found that the composition and function of gut microbiota were associated with BBIBP-CorV vaccine response: Short-chain fatty acids metabolized in the gut were positively correlated with antibody responses [19]. The above studies identified specific gut microbiota associated with improved immune responses to vaccines, providing evidence for targeting the gut microbiota to improve vaccine efficacy. Gut microbiota is not only a key factor that causes differences in immune responses to the same vaccine in different countries and regions but also an important factor that affects the different immune efficacy of the same individual to different types of vaccines. Therefore, targeting gut microbiota to optimize vaccine efficacy requires stratification according to different populations, and specific vaccines also require specific microbiota.

**Table 1:** Effect of gut microbiota on vaccine efficacy

model	host	vaccine	gut microbiota associated with vaccine efficacy	reference
Animal models	Mice	Ovalbumin	Lactobacillaceae, Rumen family, and Clostridium bacteria were associated with vaccine efficacy	[4]
	Mice	RVV	The abundance of Clostridium and Lactonemae was positively correlated with vaccine efficacy	[9]
Clinical studies	Ghanaian and Dutch infants	RV	Rotavirus vaccine responders in Ghana and the Netherlands were associated with an increased abundance of Streptococcus Bovis and decreased abundance of Bacteroides phylum; Ghanaian nonresponders had an increase in enteric streptococci and a decrease in Bacteroides	[10]
	Pakistani and Dutch infants	RV	Pakistani responders were associated with increased Streptococcus Bovis abundance and decreased Bacteroides phylum abundance	[11]
	Ghanaian infants	RV	Phage diversity and the presence of enterovirus B and multiple novel co-viruses were inversely correlated with vaccine efficacy	[12]
	Nicaraguan infants	RV	Proteus and Egella abundance were positively correlated with vaccine efficacy, and Fusobacterium and Enterobacteriaceae were negatively correlated with vaccine efficacy	[14]
	Rural Zimbabwe infants	RV	Bacteroides multiforme was associated with serum anti-rotavirus IgA titer	[15]
	Bangladeshi infants	BCG TTV HBV OPV	The abundance of Bifidobacterium longum subspecies was positively correlated with OPV, BCG, TTV, and HBV efficacy; Clostridium, Enterobacteriaceae, and Pseudomonas abundance were inversely correlated with vaccine efficacy	[16]
	New Hampshire infants	TTV	Bifidobacteria abundance was negatively correlated with vaccine efficacy, and the abundance of CDP-diacylglycerol biosynthesis pathway-associated species was positively correlated with vaccine efficacy	[17]
	HongKong adults	SARS-CoV-2 vaccine (CoronaVac and BNT162b2)	Bifidobacteria abundance was positively correlated with CoronaVac vaccine efficacy; Bacteria rich in flagella and fimbriae were positively correlated with the efficacy of the BNT162b2 vaccine; Individuals with fewer adverse events after vaccinating any of the two vaccines were enriched with large amounts of Copriprevoria and two Megamonas	[18]

RVV, rabies virus vaccine; OPV, oral polio vaccine; BCG, Bacillus Calmette-Guerin;

RV, rotavirus vaccine; TTV, tetanus toxoid vaccine; HBV, hepatitis B vaccine.

## Gut microbiota affects vaccine efficacy by modulating the immune responses

Molecules carried or derived from gut microbiota could regulate host immune responses by acting as innate immune adjuvants or inducing cross-immunity, thereby affecting vaccine efficacy [20].

### Natural immune adjuvants

The effects of vaccines are mediated by the induction of antigen-specific immune responses, however, vaccine antigens themselves are usually poorly immunogenic, thus requiring adjuvants to obtain an adequate immune response. Adjuvants could enhance immunogenicity and vaccine efficacy [1]. Parenteral and mucosal (oral) vaccines require different types of adjuvants, and choosing an appropriate adjuvant is critical as it could greatly affect the long-term protective efficacy of the vaccine. It has been shown that the gut microbiota is a constant source of natural adjuvants that could affect vaccine efficacy. For example, trivalent inactivated influenza vaccine-specific antibody titers and plasma cell frequencies in peripheral blood were reduced in germ-free or antibiotic-treated mice following the vaccination of human trivalent inactivated influenza vaccine, whereas rebuilding the gut microbiota by oral a flagella-containing E. coli would restore the vaccine efficacy. The mechanism was that binding of bacterial flagellin to Toll-like receptor (TLR) 5 induced macrophages to secrete interleukin (IL)-6, proliferation-inducing ligand, and TNF-α, resulting in increased plasma cell differentiation. TLR5-mediated microbiota sensing also affected antibody responses to the inactivated polio vaccine, but not the adjuvanted vaccine and live-attenuated yellow fever vaccine [21]. After the oral cholera vaccine, individuals with higher intestinal Clostridium abundance and lower Enterobacteriaceae abundance were more likely to enhance IgG- and IgA-secreting memory B-cell responses, which targeting the O-specific polysaccharide of Vibrio cholera. And the levels of IL-1β and IL-6 secretion by fecal-induced macrophage from higher memory B-cell response individuals were significantly different from those in less responsive individuals [22]. Stražar, M. et al. found that the gut commensal bacteria Rothia might inhibit the production of BCG-induced non-specific immune cytokines IL-6, IL-1β, and TNF-α by affecting phenylalanine metabolism, while Eggertia positively correlated with BCG-induced specific T cell-mediated memory responses [23]. These studies suggest that the gut microbiota influences the immune response to vaccines and is a good source of natural immune adjuvants.

### Regulation of immune system development and immune response

The early-life gastrointestinal microbiota is critical for the development and maturation of the infant’s mucosal and systemic immune systems [3]. For example, germ-free mice had low levels of IgA in serum and intestinal, reduced numbers of IgA-producing plasma cells, and dysplasia of Peyer’s plaques. However, when germ-free mice were colonized with commensal bacteria, IgA production would reach normal levels [24]. In germ-free or antibiotic-treated mice, in addition to the reduction of the number of IgA-producing cells, the number of phagocytes and antigen-presenting cells (such as dendritic cells, macrophages, and neutrophils) was also reduced, and with insufficient T cell differentiation, suggesting that these cells also require the stimulation of commensal bacteria [24]. Riboflavin derivatives produced by Bifidobacterium, Bacteroides thetaiotaomicron, Lactobacillus casei, and Enterobacter cloacae activated mucosa-associated T cells through restricted major histocompatibility complex-associated protein-1 [25]. In addition, short-chain fatty acids produced by gut bacterial metabolism could pass through the bloodstream, induce immune cell development in the bone marrow, and affect lung immune responses [26]. Segmented filamentous bacteria attached to the epithelial cells of the lower small intestine could not only stimulate IgA response, but also activate lamina propria dendritic cells and macrophages to secrete IL-1β, IL-6, and IL-23, and induce the generation of intestinal mucosal specific Th17 cell population, which has the potential to differentiate into RORgt subset cells [27]. Microbe-specific T cells could provide tailored signals to follicular B cells in gut-associated lymphoid tissue, thereby enhancing diversification and selective isotype switching [28]. This could be effectively combined with a vaccine to boost anti-pathogen antibody responses. These studies demonstrated that gut-specific microbiota and their derivatives could regulate immune system development and immune responses, and targeting microbiota has the potential to improve host immune responses.

### Carrying vaccine-like epitopes to induce cross-immunity

Gut microbiota could adversely affect vaccine efficacy by biasing antibody responses toward nonprotective vaccine antigens which are similar to commensal bacterial antigens. For example, cross-reactivity of pre-existing specific memory T and B cells for the human immunodeficiency virus (HIV)-1 envelope (Env) glycoprotein with gut commensal antigens might lead to antibody targeting gp41, which was a nonprotective epitope of HIV, thereby reducing the efficacy of HIV vaccine [29]. Therefore, altering the existing gut microbiota in the infant might have a beneficial effect on B cells to elicit a more functional anti-HIV antibody response. Studies on SARS-COV-2 vaccines also found that microbial proteins derived from human and mice gut commensal bacteria (such as heat shock protein 60 and heat shock protein 70 derived from Escherichia coli) were similar to the linker domain (1147- SFKEELDKYFKNHT-1160, P144) of the SARS-COV-2 protein S2, which induced reactive monoclonal antibodies could bind to S2 and P144. Mice with pre-existing high levels of S2 cross-reactive antibodies produced higher S protein-specific binding antibodies, especially antibodies against S2, after immunization with the SARS-COV-2 S DNA vaccine. Similarly, pre-existing S2- and P144-specific antibody levels were positively correlated with receptor-binding domain-specific antibody titers after vaccinating two doses of inactivated SARS-COV-2 vaccine in humans [30]. These studies suggested that gut microbiota could affect vaccine efficacy by inducing cross-immunity by carrying vaccine-like epitopes.

## Targeting the gut microbiota to modulate vaccine efficacy

Studies in humans had shown an association between better vaccine responses and specific bacterial taxa. These associations varied with different vaccine strategies, and modulation of gut microbiota through measures such as antibiotics, probiotics, engineered bacteria, etc. was considered an important way to enhance vaccine effectiveness [20].

### Antibiotics

Modulation of gut microbiota by antibiotics has a major impact on vaccine response. A randomized controlled clinical trial compared the effect of healthy adults randomized to broad-spectrum (vancomycin and ciprofloxacin and metronidazole), narrow-spectrum (vancomycin), or no antibiotics on the immune response to ORV. It was found that although the antibiotics did not change the absolute titers of anti-rotavirus IgA in the receptors sera, in the narrow-spectrum group, the immunogenicity of ORV was enhanced on day 7 after vaccination. In addition, antibiotics increased the fecal shedding of rotavirus, while also rapidly altering the diversity of gut bacteria. On day 7 post-inoculation, members of the Bacteroides phylum, especially Prevotellaceae, could serve as specific bacterial taxa to distinguish ORV enhancers from rotavirus shedders [31]. This study demonstrated that gut microbiota modification altered immune responses to ORV and supported further exploration of gut microbiota manipulation to enhance ORV immunogenicity.

### Probiotics

Beneficial modulation of the gut microbiota is an effective strategy to improve the efficacy of vaccine-induced immunity. For example, oral administration of Lactobacillus Plantarum strain GUANKE could increase the serum level of neutralizing antibodies and cellular immune responses after intramuscular injection of the SARS-CoV-2 DNA vaccine in mice [32]. The addition of Bacillus subtilis spores to an intramuscular vaccine formulation of inactivated avian influenza virus resulted in enhanced H9N2 virus-specific antibody(IgG) responses [33]. The combined use of Bacillus subtilis and the live coccidiosis vaccine could enhance the efficacy of the live coccidiosis vaccine, prevent poultry coccidiosis, improve broiler production, and prevent Eimeria infection [34]. A new vaccine using Enterococcus faecium as a bacterial vector carrying oral influenza antigens induced antigen-specific antibodies and protected mice from lethal H1N1 infection [35]. At 4 months of age, infants given an enhanced formula to promote the growth of intestinal Bifidobacteria showed enhanced oral polio vaccine-specific responses after vaccination, and the percentage of bifidobacteria in the gut microbiota was positively correlated with poliovirus IgA titers [36]. Adding a probiotic mixture (Lactobacillus Plantarum and Bifidobacterium animal and Bifidobacterium longum infantis) to the influenza vaccine, elderly receptors had enhanced total antioxidant capacity, increased β-defensin levels, and increased the abundance of health status-related gut microbiota [37]. Mice supplemented with prebiotic lactosaccharide 2’-fucosyllactose and a complex mixture of immunomodulatory prebiotic short-chain galactooligosaccharides and long-chain fructooligosaccharides in early life improved the specific antibody response of male mice to trivalent inactivated influenza vaccine [38]. These findings suggest that the incorporation of probiotic strains into vaccine components or modulation of the abundance of beneficial bacteria in the gut through prebiotics could enhance the immune efficacy of vaccines.

### Engineering bacteria

A growing body of research has shown that gut microbiota is important for both mucosal immunity and systemic immune responses to pathogens and oral vaccines. Oral vaccines that deliver human papillomavirus surface-anchored antigens through genetically modified lactic acid bacteria-induced stronger systemic and mucosal-specific cytotoxic immune responses, reducing human papillomavirus infection, and thus reducing the incidence of cervical cancer [39]. This experiment showed that the modified lactic acid bacteria could serve as mucosal vaccine carriers and could improve the immune efficacy of the delivered vaccines.

## Summary and outlook

Gut microbiota could act as a highly adaptable tissue-specific adjuvant to modulate immune responses and affect the immunogenicity and efficacy of vaccines. Targeting gut microbiota has been considered an important strategy to improve vaccine effectiveness. But current research on gut microbiota’s impact on vaccine efficacy has been largely cross-sectional, linking microbiota to vaccine response only at a certain point in time. Over time, the composition of the gut microbiota undergoes large changes in response to environmental exposures. Therefore, more longitudinal studies are needed to better assess the impact of gut microbiota on vaccine response. In the context of future vaccine trials, it might be important to stratify individuals according to their gut microbiota profile and metabolism, as well as the influence of host genetics. The current approach to vaccine development and its administration requires a major shift. For example, future vaccines could be designed to include specific immune-modulating probiotics to compensate vaccine recipients whose guts lack essential immune-stimulating microbes.

## Data Availability

Not applicable.
