# Correction: Recent five-year progress in the impact of gut microbiota on vaccination and possible mechanisms

**DOI:** 10.1186/s13099-023-00560-1

**Published:** 2023-07-10

**Authors:** Biqing Huang, Jianwei Wang, Lanjuan Li

**Affiliations:** 1 State Key Laboratory for Diagnosis and Treatment of Infectious Diseases, National Clinical Research Center for Infectious Diseases, Collaborative Innovation Center for Diagnosis and Treatment of Infectious Diseases, The First Affiliated Hospital, Zhejiang University School of Medicine, Hangzhou, China; 2 Research Units of Infectious Disease and Microecology, Chinese Academy of Medical Sciences and Peking Union Medical College, Hangzhou, China; 3 NHC Key Laboratory of Systems Biology of Pathogens and Christophe Mérieux Laboratory, Institute of Pathogen Biology, Key Laboratory of Respiratory Disease Pathogenomics, Chinese Academy of Medical Sciences and Peking Union Medical College, Beijing, China

**Correction: Gut Pathogens (2023) 15:27** 10.1186/s13099-023-00547-y

Following publication of the original article [1], it was noted that corrections to the corresponding author, affiliation information, and funding note were missed in the published version of the article.

It has been corrected in this correction.

1. The corresponding author should be Dr. Lanjuan Li only.
2. The correct affiliation for Dr. Jianwei Wang should be affiliation 3 only.
3. **Funding**
  The CAMS Innovation Fund for Medical Sciences (No. 2019-I2M-5-045)

The original article [1] has been updated.
